# Case Report: Life-threatening overlap of hemophagocytic syndrome and atypical hemolytic uremic syndrome in a patient with autoimmune polyglandular syndrome type 1 successfully treated with targeted immunotherapy

**DOI:** 10.3389/fimmu.2025.1667000

**Published:** 2025-09-09

**Authors:** Emma Coppola, Giuliana Giardino, Roberta Romano, Donatella Capalbo, Paola Italiani, Diana Boraschi, Antonio De Rosa, Elisabetta Toriello, Annateresa Palatucci, Valentina Rubino, Giuseppina Ruggiero, Mariacarolina Salerno, Claudio Pignata, Emilia Cirillo

**Affiliations:** ^1^ Department of Translational Medical Sciences, Pediatric Section, Federico II University, Naples, Italy; ^2^ Institute of Biochemistry and Cell Biology, National Research Council, Naples, Italy; ^3^ Department of Translational Medical Sciences, University of Naples Federico II, Naples, Italy

**Keywords:** autoimmune polyglandular syndrome type 1, hemophagocytic lymphohistiocytosis, atypical hemolytic uremic syndrome, inborn error of immunity, target therapy

## Abstract

**Background:**

Autoimmune polyglandular syndrome type 1 (APS-1) is a rare inborn error of immunity caused by mutations in the *AIRE* gene, typically associated with chronic mucocutaneous candidiasis, hypoparathyroidism, and adrenal insufficiency. We report the first known case of APS-1 complicated by a life-threatening combination of secondary hemophagocytic lymphohistiocytosis (sHLH) and atypical hemolytic uremic syndrome (aHUS), successfully treated with targeted and supportive therapies.

**Case report:**

A 16-year-old female with a diagnosis of APS-1 confirmed by the presence of the nonsense variant c.415C>T (R139X) in exon 3 and the Finnish major mutation c.769C>T (R257X) in exon 6 of the AIRE gene presented with fever, cytopenias, organomegaly, and hyperferritinemia, fulfilling criteria for sHLH. Despite immunosuppressive therapy, she developed acute kidney injury, thrombocytopenia, and microangiopathic hemolytic anemia, consistent with aHUS. Treatment with the IL - 1 receptor antagonist anakinra and the complement inhibitor eculizumab led to rapid resolution of systemic inflammation and progressive renal and hematological recovery.

**Conclusion:**

sHLH is an exceptionally rare complication in APS-1 and has so far been reported in only one patient with a combined EBV and SARS-CoV-2 infection. aHUS has never been described in patients with APS-1. This case highlights the potential for hyperinflammatory and complement-mediated complications in APS-1, supporting the hypothesis of a cytokine storm syndrome that bridges features of sHLH and aHUS. It broadens the known spectrum of immune dysregulation in APS-1 and underscores the importance of early recognition and combined immunomodulatory treatment in similar clinical scenarios.

## 1 Introduction

Autoimmune Polyendocrine Syndrome Type 1 (APS-1), also referred to as Autoimmune Polyendocrinopathy-Candidiasis-Ectodermal Dystrophy (APECED), is a rare monogenic disorder characterized by immune-mediated destruction of multiple endocrine organs. It typically manifests in childhood with a classic triad of chronic mucocutaneous candidiasis, hypoparathyroidism, and adrenal insufficiency (Addison’s disease). APS-1 is caused by loss-of-function variants in the *autoimmune regulator* (*AIRE*) gene, which encodes a transcription factor highly expressed in the thymus. Aire plays a critical role in central immune tolerance by promoting the negative selection of autoreactive T cells and regulating the expression and presentation of tissue-specific antigens by medullary thymic epithelial cells. A wide variability of the clinical expression, including endocrine and non-endocrine diseases, with no significant correlation between genotype-phenotype, has been reported (1–3).

Hematological and renal manifestations have also been reported in APS-1, including autoimmune cytopenia, vitamin B12 deficiency–related anemia, large granular lymphocyte (LGL) leukaemia, and interstitial nephritis. The latter is primarily driven by T-cell infiltration of the renal tubules or the presence of autoantibodies targeting proximal tubular and duct-specific antigens (4). Despite supportive care, mortality in APS-1 may exceed 30%, reflecting the severity of its systemic involvement. While significant progress has been made in managing individual autoimmune components, no current therapy effectively targets the multisystemic nature of APS-1 (5, 6).

Hemophagocytic Lymphohistiocytosis (HLH) is a life-threatening hyperinflammatory syndrome characterized by excessive activation and proliferation of lymphocytes and macrophages, leading to uncontrolled immune activation and tissue damage. The etiopathogenesis involves a failure in the regulation of the immune response, particularly impaired cytotoxic function of natural killer (NK) cells and cytotoxic T lymphocytes (CTLs). This defect results in persistent activation of immune cells and overproduction of pro-inflammatory cytokines (a “cytokine storm”), causing widespread hemophagocytosis and multi-organ dysfunction (7).

Thrombotic microangiopathy (TMA) encompasses a heterogeneous group of monogenic and acquired syndromes characterized by endothelial injury leading to widespread microvascular thrombosis, resulting in microangiopathic hemolytic anemia, thrombocytopenia, and organ dysfunction. Pathogenesis involves damage to endothelial cells lining small blood vessels, triggering platelet aggregation and microthrombi formation that obstruct blood flow. Etiologically, TMA includes a spectrum of disorders: Thrombotic thrombocytopenic purpura (TTP), primarily caused by severe deficiency of the metalloprotease ADAMTS13, leading to accumulation of ultra-large von Willebrand factor multimers and platelet clumping; Hemolytic uremic syndrome (HUS), classically triggered by Shiga toxin-producing *Escherichia coli* infection; Atypical HUS (aHUS), a complement-mediated TMA resulting from genetic mutations or acquired dysregulation of the alternative complement pathway. In aHUS, uncontrolled activation of the complement cascade, particularly the alternative pathway, leads to excessive formation of the membrane attack complex (MAC, C5b-9), causing direct endothelial cell injury. This injury promotes a prothrombotic state with platelet activation and microvascular thrombosis. Mutations in complement regulatory proteins such as factor H, factor I, or membrane cofactor protein (MCP/CD46) impair the ability to control complement activation, exacerbating endothelial damage. Secondary causes of TMA may include certain drugs, autoimmune diseases, malignancies, pregnancy, and transplantation (8, 9).

If not recognized and treated appropriately, both HLH and TMA may lead to a fatal outcome. The association between TMA and HLH has been previously described (10, 11) but never in IEI patients. In this report, we present the first documented case of a patient with APS-1 who developed a life-threatening combined secondary-HLH and TMA in the form of aHUS, successfully treated with anakinra and eculizumab.

## 2 Case report

The patient, a female adopted at the age of 6, was diagnosed as APS-1 at the age of 8. She received a standard set of vaccinations including Bacillus Calmette–Guérin during her first years of life, with poor response to tetanus and diphtheria. Before being diagnosed as APS-1, the patient suffered from recurrent otitis and pneumonia, *Giardia lamblia* infection and experienced herpes keratitis. At the age of 15 months, she was treated with standard antitubercular chemotherapy due to *Mycobacterium tuberculosis* infection. Oral candidiasis, palm-plantar and face warts were also detected.

**Figure 1:**
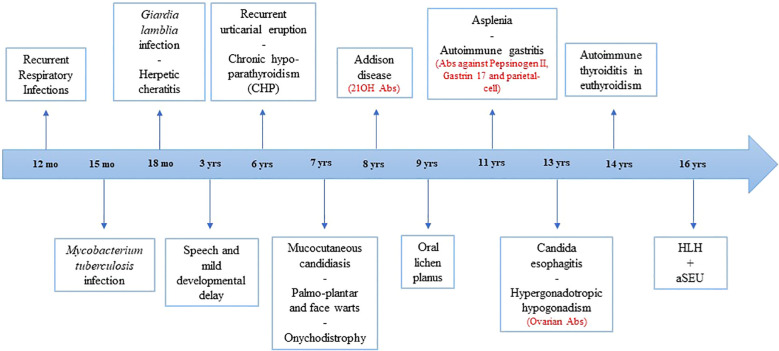
Timeline of major clinical events of patient. Positive autoantibodies are shown in red. HLH, hemophagocytic lymphohistiocytosis; aHUS, atypical hemolytic uremic syndrome; mo, months; yrs, years; Abs, autoantibodies.

At the age of 8 years, she was referred to the Federico II Pediatric Immunodeficiency Center. Proliferative response to common mitogens and immunoglobulin levels were normal. Immunophenotyping showed a normal CD4^+^ to CD8^+^ T-cell ratio with an increased number of activated HLA-DR^+^CD3^+^ T cells. High levels of serum IL-1β were detected (62.64 pg/mL; normal <3.9 pg/mL). Chronic hypoparathyroidism and Addison disease lead to the suspicion of APS-1. Sanger Sequencing of the *AIRE* gene revealed the nonsense variant c.415C>T (R139X) in exon 3 and the Finnish major mutation c.769C>T (R257X) in exon 6. Further clinical manifestations are reported in **Figure 1**. In addition, the patient also tested positive for the following circulating autoantibodies (Abs): 17α-hydroxylase (17αOH) Abs, P450 side-chain cleavage enzyme (P450scc) Abs, glutamic acid decarboxylase (GAD) Abs, anti-parietal cell antibodies (APCA), tryptophan hydroxylase (TPH) Abs, aromatic L-amino acid decarboxylase (AADC) Abs, and autoantibodies against interferon-ω (IFN-ω) and interferon-β (IFN-β).

**Figure 2:**
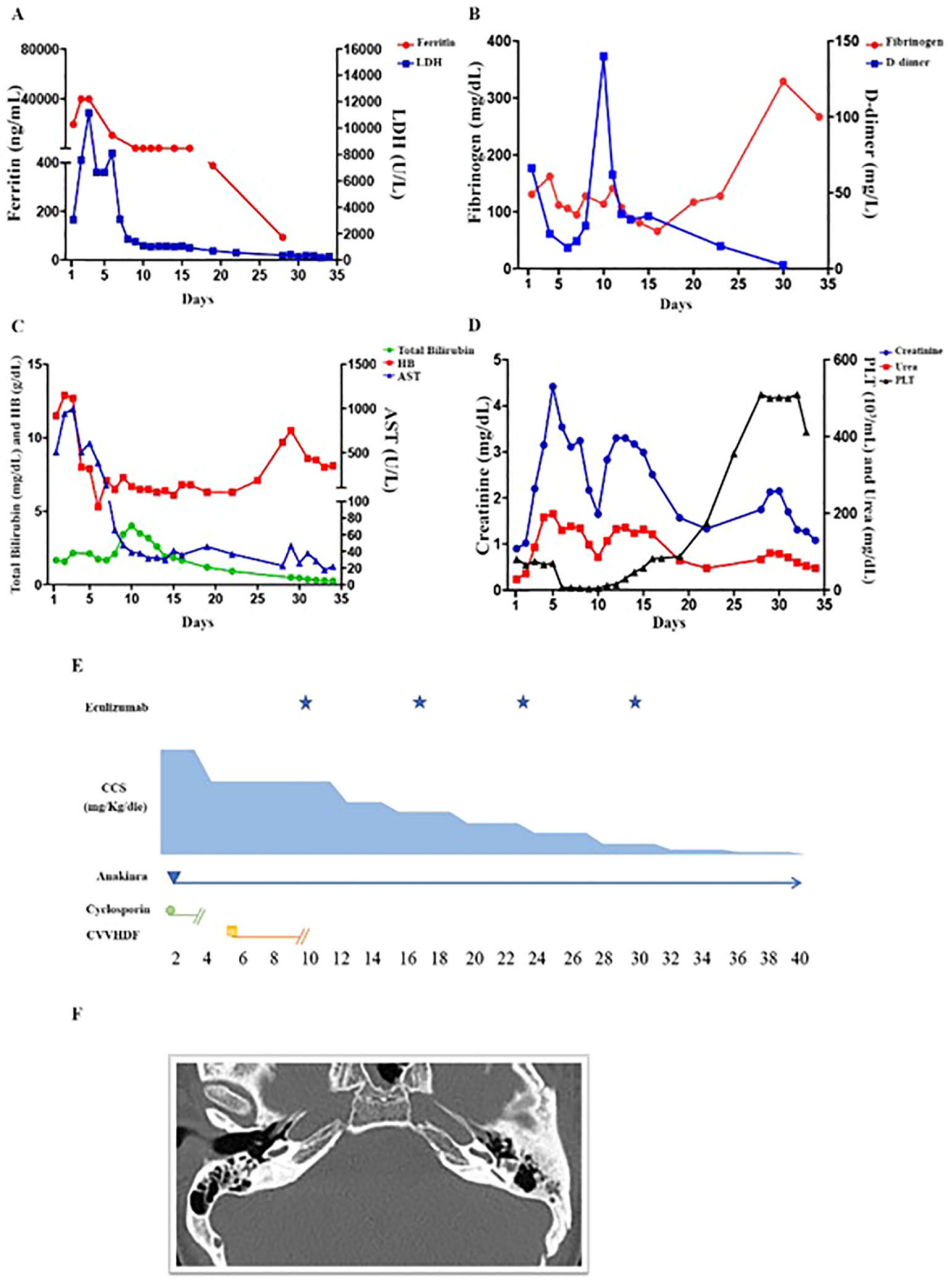
Laboratory parameters and schematic representation of the treatment in the first 40 days from the onset. **(A)** The ferritin values of day +2, +3 were greater than 40.000 U/L (upper limit of our clinical lab detection). On day +6 a significant reduction was observed and a trend to normalization was obtained on day +10 (before eculizumab). Very high levels of LDH persisted until day +6. A significant reduction was observed on day +7. **(B)** Hypofibrinogenemia required multiple fresh frozen plasma infusions. Normalization was achieved only belatedly (day +34). D-dimer serum levels decreased progressively until (day +6) due to combined effect of anakinra and of supportive care. On day +9, a further increase was observed. At this time point, a blood culture from central venous catheter revealed Candida tropicalis proliferation, which was treated with anidulafungin. **(C)** The patient received multiple RBC transfusions from day +6 to day +8 since HB dropped to 5.3 g/dl on day +6. Other evidence for intravascular hemolysis included reduced haptoglobin and serum bilirubin elevation. The resolution of hemolysis was evidenced by improvement in bilirubin and LDH levels following the first or the second dose of eculizumab, respectively. However, anemia persisted up to day +28 (Hb: 9.7g/dL), due to frequent blood draws and kidney failure. **(D)** Thrombocytopenia coincided with hyperferritinemia, elevation in AST, LDH, and preceded the elevation of total bilirubin, creatinine and plasma urea. Differently from ferritin and AST, thrombocytopenia worsened progressively reaching a nadir before eculizumab (4.000 cells/mm3 on day +10). After 3 days from the first administration, the platelet number started to increase. Normalization was obtained five days after the second dose. Creatinine and plasma urea increased after CVVHD suspension, but a trend towards reduction was observed after 4 days from the first eculizumab dose. **(E)** Schematic representation of the immunomodulatory treatment. Due to renal failure, anakinra (3.7 mg/kg/die) was given every other day from day + 4 to day +13. From day +14 to +17, the dosage decreased to 1.8 mg/kg every other day. From day +18 to +23 the patient received 0.9 mg/kg every other day. Eculizumab, 900 mg total, was initiated on day +10 and given at the same dosage every 7 days during the first month of treatment. **(F)** Ear CT scan revealing lateral semicircular canals dysplasia and apex sclerosis of the left tympanic membrane.

In September 2019, at the age of 16, she was admitted due to fever, dyspnea and diarrhea. Laboratory tests and clinical examination revealed: lymphopenia (670/µL), thrombocytopenia (81.000/µL), increased serum levels of lactate dehydrogenase (3.063 U/L), ferritin (19.637 ng/mL), aspartate aminotransferase (506 U/L), triglycerides (362 mg/dL), hypofibrinogenemia (131 mg/dl), and liver enlargement, suggesting a sHLH. A chest X-ray showed interstitial lung infiltrates and pleural effusion. Nasal, pharyngeal and rectal swab, urine, blood culture and PCR for HSV1/2, Parvovirus B19, HHV8, HHV6, EBV, CMV and other respiratory viruses in the blood and bronchial aspirate were negative. Treatment with ceftriaxone, cyclosporine and methylprednisolone was started. After 24 hours, severe abdominal pain and melena occurred. Esophagogastroduodenoscopy was normal, but biopsy was not performed due to severe coagulopathy, requiring multiple plasma infusions. Pericardial effusion, abdominal ascites and markedly thickened gallbladder with biliary sludge were also detected. Due to cyclosporine failure in the control of the hyper-ferritinemic inflammation (ferritin >40.000 ng/mL on day +2 and +3) (**Figure 2A**), and the trend to increase in serum levels of creatinine (range 1.65-2.20 mg/dl), cyclosporine was stopped and anakinra (3.7 mg/kg/die) was started, resulting in disappearance of fever by 24 hours along with a clear-cut decrease of the major inflammatory markers (**Figures 2A–C**).

On day +5 from the admission, creatinine serum levels significantly raised (4.22 mg/dL; normal range 0.57-1.11 mg/dL) and estimated glomerular filtration rate was 16.05 mL/min/1.73 m2 (normal range 112±13 mL/min/1.73 m^2^). Urine biochemistry showed microhematuria (2625/µL; normal range 0-14) and proteinuria (402 mg/dL/24h). The patient became oliguric and was admitted to the intensive care unit on day +6. Initial treatment comprised five sessions of renal replacement therapy through continuous veno-venous hemodiafiltration (CVVHDF), five sessions of plasmapheresis, non-invasive positive pressure ventilator support, empiric antibiotic and antifungal therapy. Improvement of respiratory dynamic was achieved. Despite this, no beneficial effect was observed in platelet counts which dropped from 75.000 to 7.000/µL (**Figure 2D**), along with worsening of anemia (Hb nadir: 5.3 g/dl), increase in serum levels of bilirubin (4.02 mg/dl) and persistence of high levels of lactate dehydrogenase. Low levels of haptoglobin, negative Coombs test and the presence of schistocytes were congruent with a diagnosis of TMA. aHUS was confirmed since ADAMTS13 activity was normal (>10%). Immunological tests demonstrated low serum levels of C3 (0.43mg/dL; normal 0.51-0.95). Rheumatoid factor, antinuclear, extractable nuclear, perinuclear anti-neutrophil cytoplasmic and proteinase 3 anti-neutrophil cytoplasmic antibodies were all negative. Testing for anti-CFH antibodies was negative, excluding autoantibody-positive HUS. Serum levels of homocysteine and methylmalonic acid were normal, thus cobalamin C deficiency was ruled out.

The recombinant human monoclonal anti-C5 antibody eculizumab (900 mg) was initiated on day+10 (**Figure 2E**). A progressive nephrological and hematological improvement was achieved (**Figure 2D**).

Next-generation sequencing, covering approximately 5,000 genes, including those associated with familial HLH, and wide range of IEI and complement deficiencies (such as C3, CFB, CFH, CFHR1, CFHR3, CFHR5, CFI) and MLPA analysis for CFHR1/CFHR3 deletions, was performed. No pathogenic mutations were identified, except for a single heterozygous variant, c.4016G>A, in the LYST gene, which was classified as likely benign.

NK-cell activity, measured by incubating PBMNc with K562 targets cells for 4 hours at an effector-target (E/T) cell ratio and NK-cell degranulation following stimulation with K562 cells were slightly diminished (E/T ratio 1:1, 5.4 vs 18.8% in the ctr; CD56^+^CD3^-^CD107a^+^ 40.5 vs 53.5% in the ctr, respectively).

The patient remained on eculizumab for 24 months, as the initial presentation was severe and the risk of further TMA manifestations was deemed too great. During immunosuppression, terminal complement complex activation evaluated by measuring the level of sC5b-9 in blood, renal functionality and hematological findings were found persistently normal. Eculizumab was then stopped and, at the latest follow-up, 5 years from sHLH/aHUS, the patient is well with no further events.

By day +9, the patient developed persistent bilateral sensorineural hearing loss. CT and MRI of brain and of the ears revealed dysplasia of the lateral semicircular canals and apical sclerosis of the left tympanic membrane, findings suggestive of an underlying chronic inflammatory process. The internal auditory canals and inner ear structures appeared normal (**Figure 1F**). Serological tests for antibodies against cochlear antigens and the glomerular basement membrane were negative. Given the context of aHUS-associated thrombosis and the suspicion of an ischemic event superimposed on a preexisting chronic condition, the patient underwent prolonged hyperbaric oxygen therapy. However, due to lack of clinical response and persistent hearing loss, bilateral hearing prostheses were subsequently implanted.

## 3 Discussion

Secondary Hemophagocytic Lymphohistiocytosis (sHLH) has been reported as an initial manifestation in several inborn errors of immunity (IEI). It can be triggered by various infections, with DNA viruses such as Epstein-Barr virus (EBV), cytomegalovirus (CMV), and adenovirus being the most common recognized agents. Patients with APS-1 are generally not more susceptible to infections beyond chronic mucocutaneous candidiasis (CMC). However, a subset of APS-1 patients may develop recurrent or severe infections caused by herpesviruses like herpes simplex virus (HSV) and varicella-zoster virus (VZV) (6). More recently, an increased risk of severe pneumonia following SARS-CoV-2 infection has been described in APS-1 patients due to pre-existing neutralizing autoantibodies against type I interferons (IFNs) (12). Additionally, APS-1 patients with asplenia face a higher risk of invasive infections by encapsulated bacteria. Our patient, in addition to the expected candidiasis and HSV-related keratitis, has a history of infection with *Mycobacterium tuberculosis* and *Giardia lamblia*. However, despite asplenia, she has not experienced any severe infections caused by encapsulated bacteria.

HLH is an exceptionally rare complication in APS-1 and has been reported only once previously, in a patient with simultaneous EBV and SARS-CoV-2 reinfection (13). On the other hand, aHUS has never been described in APS-1 to date.

Although sHLH and aHUS appear as distinct and sequential clinical entities, they may represent different facets of a broader pathological process known as cytokine storm syndrome (CSS). This is supported by the coexistence of multiorgan failure accompanied by coagulopathy, complement activation (complementopathy) and endothelial dysfunction (endotheliopathy) (5, 14). The observed clinical improvement with therapies targeting both cytokine neutralization and C5 complement blockade further substantiates this link.

CSS arises from an inability of the immune system to restore homeostasis after activation triggered by infections, tumors, iatrogenic factors, or underlying monogenic and autoimmune disorders. Central to the initiation and perpetuation of CSS are pro-inflammatory cytokines including interleukin (IL)-1, IL-6, interferon-gamma (IFN-γ), and IL-18. These cytokines activate intracellular signaling pathways that induce transcription of pro-inflammatory genes and modulate the activity of both innate and adaptive immune cells.

IL-1 has a central upstream role in the cytokine cascade, driving inflammation through both IL-1α, released by innate immune cells, and IL-1β, which requires inflammasome-mediated activation. Both isoforms promote further cytokine production (e.g., IL-6, TNF-α) and immune cell recruitment (Th1, Th17) and the transcription of inflammatory genes (15). The role of IL-1 in CSS, including sepsis-related and autoimmune-associated sHLH, is well established, supporting the therapeutic use of anakinra (14, 16–20).

In our case, the patient showed a rapid clinical and laboratory response to anakinra within 24 hours, in line with elevated baseline IL-1 levels, suggesting a key role for IL-1 in the hyperinflammatory process. Given the rapid clinical improvement following anakinra, it was not necessary to escalate to other therapeutic strategies used in refractory cases of HLH, such as emapalumab (a monoclonal antibody targeting IFN-γ) or JAK inhibitors (16).

Complement activation plays a critical role in CSS pathogenesis. In particular, components such as C3a, C5a, and the membrane attack complex (C5b-9) are excessively generated, amplifying inflammation and endothelial injury. This mechanism mirrors the pathogenesis of aHUS, where dysregulation of the alternative complement pathway, often due to genetic mutations or acquired defects in regulatory proteins like factor H, factor I, or membrane cofactor protein (MCP/CD46), leads to uncontrolled complement activation. The resultant complement-mediated endothelial damage triggers platelet activation and microvascular thrombosis, hallmark features of aHUS (5, 21).

In our patient, no infectious trigger was identified, yet an abnormal immune response to a pathogen remains the most plausible explanation. APS-1 patients lack described mechanisms for spontaneous cytokine release in the absence of a trigger, unlike other hyperinflammatory syndromes such as Castleman disease. Furthermore, other known triggers like malignancies or autoimmune flares were absent both at presentation and during follow-up. One possible mechanism involves autoimmunity triggered by molecular mimicry between a pathogen, no longer detectable at diagnosis, and self-antigens, analogous to observations in children with multisystem inflammatory syndrome following SARS-CoV-2 infection. Although no active infection was identified at the time of sHLH/aHUS diagnosis, it is noteworthy that prior exposure to *Mycobacterium tuberculosis* and other infections may have contributed to the patient’s subclinical immune dysregulation. In particular, *M. tuberculosis*, especially during latent infection, can exert chronic effects on the immune system through various mechanisms that reinforce each other, such as the modulation of programmed cell death pathways and the elimination of infected cells. These processes can lead to the release of self-antigens, which in turn activate antigen-presenting cells and promote the secretion of cytokines such as IL-1, as well as the expansion of autoreactive T cell clones (22, 23).

We also observed a mild reduction in NK cell function in our patient. No genetic substrate responsible for this alteration was identified, supporting the hypothesis of a secondary dysfunction related to the inflammatory state. In sHLH, reduced NK cell and/or CTL cytotoxicity is a key factor in the pathogenesis and progression of severe cases, although the underlying mechanisms remain incompletely understood. Recent studies have demonstrated that elevated levels of pro-inflammatory cytokines can influence NK cell activity, and in cases of sHLH, there is an alteration in the balance between activating and inhibitory surface receptors on NK cells, with an upregulation of the latter (24–26). In our case, the impaired functionality of NK cells may have compromised their cytotoxic elimination of infected or activated antigen-presenting cells, thereby contributing to the progression of immune activation and the perpetuation of the inflammatory state.

This case underscores the importance of vigilant monitoring of APS-1 patients for signs of sHLH and complement pathway activation. In cases of persistent or unexplained fever in patients with APS1, early identification of those at risk for hyperinflammatory complications is crucial. This can be achieved by assessing biomarkers such as ferritin, triglycerides, fibrinogen, as well as CRP and soluble IL-2 receptor (sCD25). Additionally, early signs of complement activation should be evaluated, including reductions in C3 levels or increases in soluble C5b-9. Recognizing the presence of sHLH and/or aHUS, as well as their potential overlap within the spectrum of CSS, is essential for prompt diagnosis and the timely initiation of targeted therapies. This approach aims to rapidly address two potentially life-threatening conditions, thereby reducing associated morbidity and mortality.

## 4 Patient perspective

Throughout the clinical course, the patient and her family received continuous, multidisciplinary support from a dedicated team of pediatric immunologists and endocrinologists. The parents were thoroughly informed about the complexity of the case and were regularly updated on the evolving clinical picture, associated risks, and available therapeutic strategies. They expressed full satisfaction with both the appropriateness of the medical management and the clinical outcomes, including their daughter’s complete recovery. The patient remains under ongoing specialist follow-up for long-term management of her underlying condition.

## Figures and Tables

**Figure:**
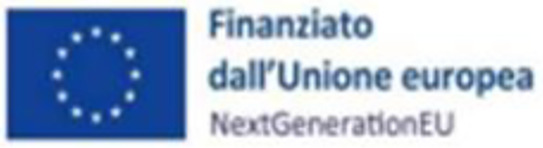


## Data Availability

The raw data supporting the conclusions of this article will be made available by the authors, without undue reservation.
